# Progress and prospects for engineered T cell therapies

**DOI:** 10.1111/bjh.12981

**Published:** 2014-06-17

**Authors:** Waseem Qasim, Adrian J Thrasher

**Affiliations:** 1 Molecular & Cellular Immunology, Institute of Child Health, University College LondonLondon, UK; 2 Great Ormond Street Hospital TrustLondon, UK

**Keywords:** transplantation, T cells, gene therapy

## Abstract

Proof-of-concept studies have demonstrated the therapeutic potential of engineered T cells. Transfer of recombinant antigen-specific T cell receptors (TCR) and chimaeric antigen receptors (CARs) against tumour and viral antigens are under investigation by multiple approaches, including viral- and nonviral-mediated gene transfer into both autologous and allogeneic T cell populations. There have been notable successes recently using viral vector-mediated transfer of CARs specific for B cell antigens, but also reports of anticipated and unanticipated complications in these and other studies. We review progress in this promising area of cellular therapy, and consider developments in antigen receptor therapies including the application of emerging gene-editing technologies.

Whilst the application of tyrosine kinase inhibitors and antibody-based therapies has become rapidly established in haematological practice (Katsnelson, 2013), the use of cell-based therapies, particularly genetically engineered T cells, is still emerging. In recent months there have been a number of encouraging reports detailing the therapeutic application of genetically modified T cells. In particular, promising data from trials investigating T cells modified to express chimaeric antigen receptors (CARs) against B cell antigens have generated extensive interest from clinicians, researchers, pharmaceutical companies and investors (Humphries, 2013). Here we review progress in this field, consider the technologies involved and how these approaches are likely to be applied, and the hurdles faced in delivering cell and gene therapies.

The potency of T cell-mediated immunity against viruses and certain malignancies is now well established. In the haematological setting, adoptive transfer of T cells against viral pathogens, such as Epstein Barr Virus (EBV), Cytomegalovirus (CMV) and Adenovirus, has been of proven benefit in the allogeneic stem cell transplant (allo-SCT) setting (Walter *et al*, 1995; Heslop *et al*, 1996, 2010; Peggs *et al*, 2003, 2011; Cobbold *et al*, 2005; Feuchtinger *et al*, 2006; Leen *et al*, 2009; Qasim *et al*, 2013a). Allogeneic T cells have been shown to mediate graft-versus-leukaemia (GvL) effects following allo-SCT, but these effects are offset by the risk of graft-versus-host disease (GVHD) (Ho & Soiffer, 2001). Approaches to mitigate against GVHD include expansion of T cell lines following antigenic stimulation with the aim of producing antigen-specific populations while diminishing alloreactive clones. This can take several weeks of culture but has been used to successfully treat viral reactivation after transplant, and more recently to treat leukaemic relapse (Chapuis *et al*, 2013). An alternative approach to circumvent the need to culture, grow and enrich T cells in this manner is to redirect T cell specificity through the transfer of antigen receptor genes. These strategies may involve recombinant variants of conventional heterodimeric αβ T cell receptor chains (TCR), or hybrid composites of antibody-like receptor chains linked to transmembrane and activation domains (CARs). Whereas αβTCRs have lower affinities and recognize processed peptide antigen expressed in the context of major histocompatibility complex (MHC), CARs are high affinity, MHC-unrestricted and capable of engaging target cell surface proteins independently of presentation pathways (Sadelain *et al*, 2013). Engineering T cells to express these receptors has harnessed gene therapy technology, which enables durable gene expression following viral vector-mediated chromosomal integration of transgenes. Similar gamma-retroviral (γRV) and lentiviral (LV) platforms have also been used to engineer T cells for a variety of additional strategies. For example, there is now two decades of experience using γRV to express prodrug-activated suicide gene mechanisms derived from Herpes Simplex Thymidine kinase (HSVTK) as a contingency against GVHD, allowing elimination of mismatched T cells causing undesirable effects after allogeneic transplantation through administration of ganciclovir (Bonini *et al*, 1997). Preclinical studies have also shown that T cells can be modified to resist agents such as ciclosporin (Brewin *et al*, 2009), potentially allowing preservation of selected functional responses during periods of post-transplant immunosuppression and emerging technologies are exhibiting remarkable efficacy in targeted gene disruption.

In this article we update progress in the application of genetically modified T cells. The technology and processes involved in genetically modifying cells is reviewed, and recent experience from early phase clinical trials summarized, including anticipated and unanticipated adverse effects. Finally, prospects for the wider application of existing approaches are considered and emerging novel technologies for gene editing and the targeted disruption of key receptors reviewed.

## Genetic modification of T cells

T cells have been attractive targets for gene modification for almost three decades, being relatively easy to harvest and culture *ex-vivo* but researchers have encountered a variety of hurdles. T cells are hard to transfect, and given their mitotic properties, stable chromosomal integration of therapeutic genes is required for sustained effects. Non-viral gene transfer strategies have proven inefficient and require months of cell culture and co-expression of drug selection genes to produce usable yields of modified cells. In contrast, replication defective γRV derived from murine Moloney leukaemia virus have been used to transduce T cells since the early 1990's (Rosenberg *et al*, 1990; Ferrari *et al*, 1992; Mavilio *et al*, 1994) with vectors devoid of packaging or envelope genes but retaining intact retroviral long terminal repeat (LTR) promoter and enhancer elements. These regions have been linked to insertional mutagenesis in gene therapy trials of inherited immune disorders where autologous haematopoietic stem cells (HSC) were transduced *ex-vivo* and reinfused. In severe combined immunodeficiency (SCIDX1) (Hacein-Bey-Abina *et al*, 2003, 2008; Howe *et al*, 2008), Wiskott-Aldrich Syndrome (Braun *et al*, 2014), and Chronic Granulomatous disease (Ott *et al*, 2006); a number of subjects subsequently developed T cell or myeloid leukaemias. However, there have been no reports of similar genotoxicity in any clinical study manipulating differentiated lymphocytes, and T cells have been shown to resist retroviral-mediated transformation (Newrzela *et al*, 2008). Data from longer term follow up studies have tracked engineered T cells for over a decade in patients and found no evidence of transformational effects (Recchia *et al*, 2006; Scholler *et al*, 2012) and in extensive pre-clinical modelling, there has only been a single report of T cell transformation in a murine T cell study using γRV (Heinrich *et al*, 2013). As well as γRV, alternative LV vector systems, derived from attenuated forms of human immunodeficiency virus (HIV)-1 (Naldini *et al*, 1996; Dull *et al*, 1998; Demaison *et al*, 2002; Qasim *et al*, 2007) are now being applied for T cell engineering. These vectors are generally considered less likely to mediate genotoxicity (Montini *et al*, 2009) and can be designed to incorporate elements that increase titre and gene expression (Dull *et al*, 1998; Zufferey *et al*, 1999; Demaison *et al*, 2002). LV vectors can carry larger transgene cargos, are capable of stably transducing minimally activated T cells and preferentially integrate in active genes, without the bias towards transcription start sites exhibited by γRV (Schroder *et al*, 2002).

One current advantage of γRV systems is the availability of stable vector packaging cell lines (Miller & Buttimore, 1986), allowing for ready scalability of production, whereas LV stocks are produced transiently, by transfection of multiple plasmids into permissive cell lines. While LV studies are now dominating gene therapy studies targeting HSC, it is likely that both platforms will continue to be exploited for T cell modification in the foreseeable future. Other viral vector systems have had limited application for T cell therapies, but Adenovirus has been used for transient episomal gene expression sufficient for T cell modification, as recently reported in a study engineering T cells to resist HIV-1 (Tebas *et al*, 2014). One alternative non-viral vector system, derived from mobile genetic elements and capable of chromosomal integration, is based on transposon transposition. Sleeping beauty (Ivics *et al*, 1997) and PiggyBac (Ding *et al*, 2005) transposons have been engineered to allow cut and paste chromosomal integration of antigen receptor genes (Ivics & Izsvak, 2011). Therapeutic genes can be delivered by electroporation in plasmid form, flanked by inverted repeat (IR) and direct repeat (DR) sequences, which are then targeted by the sleeping beauty transposase enzyme (delivered in *trans* via a second plasmid). Excision and transposition of the IR/DR flanked region results in non-biased insertion of the sequence into genomic TA dinucleotide repeat sites. The strategy has been compared to LV transduction of T cells (Field *et al*, 2013) and could provide a more cost-effective and streamlined approach to manufacturing, and the Sleeping Beauty system has recently been used in early phase trials of CAR19 T cell trials (see below) (Singh *et al*, 2014). Additional recent developments include transfer of therapeutic genes in mRNA form, using the rationale that in certain situations, transient expression of therapeutic genes in T cells may be sufficient for useful effects (Zhao *et al*, 2006). For example, transient expression of DNA nucleases allows receptor genes to be disrupted (see below) and non-sustained antigen-specific CARs may be sufficient to reduce disease burden in some conditions with a reduced risk of prolonged cytokine storms or unexpected side effects.

Quiescent T cell are relatively refractory to genetic modification and a variety of activation reagents have been used to induce T cell division, including phytohaemagglutinin, OKT3 and magnetic beads conjugated with anti-CD3 and anti-CD28 antibodies. Culture media are usually supplemented with human serum and cytokines (usually interleukin-2, IL2) and are capable of supporting T cell cultivation for several weeks. Starting material may comprise autologous or human leucocyte antigen (HLA) matched donor lymphocytes collected by venesection or by non-mobilized peripheral blood harvest. Whilst some protocols include steps to enrich CD4^+^ or CD8^+^ T cell subsets, or deplete CD4^+^ CD25^+^ regulatory T cells, activation anti-CD3/anti-CD28 results in accumulation of CD3 T cells to above 90% of cultures within 48–72 h and is currently the favoured method of activation. Transduction, bead removal and enrichment (where required) of engineered T cells under good manufacturing practice conditions is possible within the following 5 d, and readily yields sufficient cells for infusion of over 10^7^ cells/kg. Greater yields can be recovered following extended culture periods of 2 weeks or longer, but cells become prone to exhaustion and apoptosis. Some trials have aimed to engineer virus-specific T cells (Cruz *et al*, 2013) in which populations of T cells undergo active expansion in response to challenge with autologous presenting cells expressing viral antigens, although the process can take several weeks or months.

Recent studies have investigated the optimal phenotype of T cell populations for the most effective immunotherapy. Effector memory cells can dominate cultures of transduced cells, and exhibit reduced *in vivo* persistence (Berger *et al*, 2008). Longer-term persistence and function is provided by central memory phenotype T cells, and naïve T cells retain longer telomeres and the greatest proliferative potential (Hinrichs *et al*, 2011). Recently, memory stem T cells have attracted interest as populations with the greatest potential of replication and expansion (Gattinoni *et al*, 2011). Targeted transduction of these populations may offer an effective, reduced cost approach to producing engineered T cells products. Whereas some early studies have aimed to infuse billions of engineered T cells following extended *ex-vivo* culture and expansion, more recent reports (Table I) suggest dosing in the range of 10^6^–10^7^/kg may be sufficient for therapeutic effect with reduced risk of infusion-related toxicities. Importantly, experience of infusing allogeneic virus-specific T cells against CMV and Adenovirus suggest that as few at 10^4^ CD3 T cells/kg undergo *in vivo* expansion and are sufficient to clear pathogens (Peggs *et al*, 2011; Qasim *et al*, 2013a).

**Table I:** Recent gamma-retroviral and lentiviral trials of CAR T cell therapy against CD19.

Centre	Vector and CAR configuration	Patients/disease	Conditioning, dosing	Complications	Outcome	References
UPENN	LV 4-1BB, CD3ζ	3 CLL	Autologous with conditioning Cyclophosphamide Pentostatin Bendamustine Dose:10^5^–10^7^/kg	Tumour lysis syndrome	2 CR, 1 PR	Grupp *et al* (2013) Kalos *et al* (2011) Porter *et al* (2011)
		2 ALL	Autologous 1 conditioned Cyclophoshamide Etoposide 1 unconditioned Dose: 10^6^–10^7^/kg	Cytokine release syndrome B cell aplasia	2 CR, 1 durable	
NCI	RV CD28, CD3ζ	8 B lymphomas	Cylophosphamide Fludarabine Dose: 10^6^–10^7^/kg	Tumour lysis	6 remission	Kochenderfer *et al* (2013) Kochenderfer *et al* (2012) Kochenderfer *et al* (2010)
		10 post-SCT	Allogeneic post-HSCT Unconditioned	Hypotension Fever B cell aplasia	3 regression	
MSKCC	RV CD28, CD3ζ	8 CLL 1 ALL	Unconditioned or Cyclophosphamide Dose: 10^6^–10^7^/kg	Death B cell aplasia	1 response	Brentjens *et al* (2013) Brentjens *et al* (2011) Davila *et al* (2014)
		5 ALL	Cyclophosphamide Dose: <3 × 10^6^/kg	Cytokine release syndrome	5 MRD	
Baylor	RV CD28, CD3ζ	8 allo-SCT	Unconditioned		2 responses	Cruz *et al* (2013)
	RV CD3ζ CD28, CD3ζ	6 NHL	Unconditioned Dose: <10^9^/m^2^		2 responses	Savoldo *et al* (2011)

UPENN, University of Pennsylvania; NCI, National Cancer Institute; MSKCC, Memorial Sloan Kettering Cancer Center; CAR, chimaeric antigen receptor; LV, lentiviral; RV, retroviral; CLL, chronic lymphocytic leukaemia; ALL, acute lymphoblastic leukaemia; NHL, non-Hodgkin lymphoma; SCT, stem cell transplantation; HSCT, haemopoietic stem cell transplantation; CR, complete response; PR, partial response; MRD, minimal residual disease.

## Redirecting T cells to express αβ antigen-specific receptors

**Fig 1:**
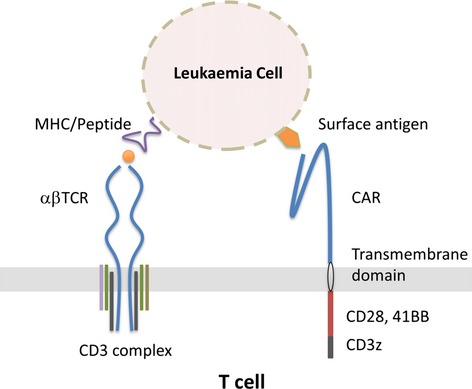
T cells engineered to express antigen-specific receptors can recognize and eliminate leukaemic cells. The αβ T cell receptor (TCR) comprises a heterodimer that can be engineered to prevent cross-pairing with endogenous TCR chains, and is expressed in association with signalling chains of the CD3 complex. TCRs recognize specific antigenic peptides that have been processed and presented in association with human leucocyte antigen (HLA) molecules expressed on the surface of target cells. Chimaeric antigen receptors are hybrid composites derived from the antigen binding domains of antibodies, linked to CD3ζ activation domains and costimulatory moieties via transmembrane bridges. These receptors mediate high affinity binding of target cell surface proteins and are independent of HLA presentation. CAR, chimaeric antigen receptor; MHC, major histocompatibility complex.

T cells recognize antigenic peptides in the context of HLA molecules via highly diversified αβ heterodimeric TCR, with CD4 T cells recognizing MHC class 1 and CD8 T cells interacting with MHC class II-presented peptide (Fig1). The TCR approach is limited to settings where TCR receptors against specific tumour antigen petide/HLA-combinations have been isolated from antigen-specific T cell clones. Retroviral transfer of genes encoding both the α and β TCR chains for a receptor specific for the melanoma antigen MART1 was the first to show efficacy in man. Engineered autologous T cells mediated anti-tumour effects in clinical trials of melanoma, with tumour regression in 2/15 subjects treated (Morgan *et al*, 2006). Subsequent application of a higher affinity TCR against MART1 resulted in responses in 6/20 patients but anticipated ‘on-target’ anti-melanin complications in skin, eyes and hair were encountered (Johnson *et al*, 2009). Side effects were also reported following infusion of carcinoembryonic antigen-specific TCRs for colorectal carncinoma, where bowel inflammation developed after therapy (Parkhurst *et al*, 2011). Minor toxicity was reported in another study that targeted the cancer-testis antigen NY-ESO-1 with notable tumour regression in patients with melanoma and synovial cell carcinoma (Robbins *et al*, 2011). In the haematological setting, very few protein antigens have proven suitable as targets for specific immunotherapy. While common mutagenic proteins such BCR-ABL1 yield few useful HLA-associated epitopes, certain otherwise normal proteins may be aberrantly expressed on tumour cells. For example, Wilms tumour antigen 1 (WT1) is expressed in leukaemic cells including acute myeloid leukaemia (AML) and myelodysplastic syndromes (MDS), at much higher levels than in normal stem cells or other tissues. Clinical trials of TCRs directed against HLA-A2/WT1 are being planned to treat relapse of AML/MDS in centres in Japan (Ochi *et al*, 2011), the USA (Schmitt *et al*, 2013) and the UK (Xue *et al*, 2005) The approach is supported by data from a recent experience with (non-engineered) donor-derived WT1-specific CD8 T cells infusions in post-transplant patients where antileukaemic effects were detected without notable toxicities (Chapuis *et al*, 2013).

A similar approach is being used to transduce donor-derived T cells from CMV seronegative stem cell donors with a HLA-A0201-restricted/CMV pp65-specific T cell TCR with a view to treating transplant recipients who reactivate CMV following the procedure. The strategy is being developed to treat other viral complications including EBV (Hart *et al*, 2008), and has also been used to engineer autologous T cells against Hepatitis B antigen for a patient with virus-related hepatocellular carcinoma (Qasim *et al*, 2013b). These approaches are attractive in mimicking native, physiological TCRs with known activity against particular antigens, and risks of aberrant effects through cross pairing of receptor chains with endogenous receptors have been mitigated by introducing additional disulphide bridges and the use of murine constant chain domains in combination with human TCR variable regions. However, the approach is constrained in being HLA-restricted, and only a limited number of tumour-specific antigens have been defined. Strategies to modify TCR complimentarity determining regions (CDRs) to enhance the affinity and avidity of αβTCRs may address some of these limitations, albeit with undefined, and difficult to predict, risks of both ‘on-target’ and ‘off-target’ side effects in normal host tissue. Recent trials of a HLA-A01 restricted, affinity enhanced MAGE-A3-specific TCR uncovered serious toxicity with unexpected recognition of titin, a cardiac petide antigen in subjects being treated for melanoma and myeloma (Cameron *et al*, 2013; Linette *et al*, 2013). These complications arose within days of therapy and despite preclinical screening for possible cross-recognition of peptide sequences and *in vitro* and *in vivo* toxicology experiments. Similarly, unanticipated on-target neural complications have arisen because of unappreciated MAGE expression in the central nervous system (Morgan *et al*, 2013).

## T cells modified to express chimaeric antigen-specific receptors

Chimaeric antigen receptors generally comprise an extracellular antibody-derived single chain antigen-recognition domain linked to a transmembrane anchor and an intracellular signalling tail (Fig1). The affinity of antigen binding afforded by antibody-derived recognition is many fold stronger than that mediated by conventional αβTCRs, and CARs have the advantage of not being HLA-restricted or dependent on antigen processing and presentation (Table II). However, only a limited number of tumour-specific targets have been defined and chimaeric receptors are non-physiological and potentially immunogenic. Transmembrane regions have been derived from CD4 or CD8 and activation regions from elements of CD3ζ in combination with CD27, CD28, 4-1BB, OX40 or other costimulatory domains (reviewed recently) (Sadelain *et al*, 2013). These receptors have evolved over three decades from first generation, murine-derived receptors, to complex third generation configurations with humanized sequences which exhibit sustained *in vivo* persistence and antitumour activity against B cell malignancies (Table I), neuroblastoma, (Park *et al*, 2007; Pule *et al*, 2008; Louis *et al*, 2011) ovarian (Kershaw *et al*, 2006) and renal carcinomas (Lamers *et al*, 2011). The consequences of introducing additional costimulatory domains into second generation CARs have been directly compared against first generation configurations in subjects with B cell lymphomas who were infused with two autologous CD19-specific CAR-modified therapies. One included both a costimulatory CD28 and the CD3ζ activation domain, while the other encoded only the CD3ζ domain. The second generation configuration supported longer *in vivo* persistence and responses in 2/6 subjects with non-Hodgkin lymphoma (Savoldo *et al*, 2011). Preclinical data comparing different constimulatory configurations suggested that 4-1BB signalling domains exhibited a reduced propensity to trigger IL2 and tumour necrosis factor α (TNFα) secretion (and thus precipitate cytokine release syndromes) compared to CD28 signalling domains (Milone *et al*, 2009). This has been supported by early clinical data from studies using the 4-1BB regions where there has been has been a suggestion of favourable clinical outcomes (Kalos *et al*, 2011) but direct comparisons in individual patients are lacking.

**Table II:** Comparison of TCR and CAR antigen-specific receptors.

Recombinant αβ TCR	Chimaeric antigen receptors
Cloned human TCR α and β variable regions Human or murine constant regions Additional disulphide bridge to prevent cross pairing with endogenous chains	Antibody-derived variable and constant regions Composite spacer, transmembrane and signalling domains
Recognize processed peptide in context of HLA	Recognize cell surface proteins independently of MHC processing or presentation
Low affinity binding	High affinity binding
Physiological signalling via the endogenous CD3 complex	Non-physiological, ectopic, CD3ζ activation

MHC, major histocompatibility complex; TCR, T cell receptor; CAR, chimaeric antigen receptor; HLA, human leucocyte antigen.

Overall, targeting cell surface marker molecules expressed on the majority of B-cell malignancies (and absent on unrelated tissues) has yielded the most promising clinical results. A number of early trials used electroporation to deliver plasmids encoding first generation anti-CD20 CARs with CD3ζ activation domains linked to antibiotic resistance/drug selection genes. Extended *ex-vivo* culture (for several months) was required to generate target cells doses in excess of 10^9^/kg, which were administered after conditioning with cyclophosphamide or fludarabine (Till *et al*, 2008; Jensen *et al*, 2010). Use of γRVs expressing second-generation anti-CD19 CARs with CD3ζ and CD28 activation domains mediated clinical responses in 6/8 patients with B cell malignancies (including chronic lymphocytic leukaemia [CLL] and follicular lymphoma) (Kochenderfer *et al*, 2010, 2012). The trial used OKT3 (anti-CD3) and IL2 for autologous T cell activation and cultured cells for over 3 weeks, before administration of cells doses up to 3 × 10^7^/kg following lymphodepletion with cyclophosphamide or fludarabine. The same group also published data from 10 subjects who received infusions (without conditioning) of up to 7·8 × 10^6^ cells/kg allogeneic donor T cells following allo-SCT for a variety of B cell malignancies that had persisted after conventional donor lymphocyte infusions. Three patients had disease regression and no patients developed GVHD although B cell aplasia was reported (Kochenderfer *et al*, 2013). Similarly modified allogeneic T cells were also used in a trial reported by researchers at Baylor, where eight subjects received cells transduced and cultured following activation by co-culture with EBV-transformed lymphoblastoid cell lines (Cruz *et al*, 2013). Again, there was no problematic GVHD but a number of subjects developed B cell lymphopenia. In the autologous setting, Memorial Sloan Kettering Cancer Center researchers have investigated similar second generation configurations, using gRV and *ex-vivo* transduction following activation with anti-CD3/28 beads, and included pre-conditioning with cyclophosphamide in some patients (Brentjens *et al*, 2011, 2013). A number of responses were documented and B cell aplasia reported. Some of the most promising data to date has been published by investigators at the University of Pennsylvania. Here, LV delivery of second generation CARs (with CD3ζ and 4-1BB domains) was combined with patient conditioning with combinations of cyclophosphamide, bendamustine, penotatsin and etoposide in patients with CLL and acute lymphoblastic leukaemia (ALL) (Kalos *et al*, 2011; Porter *et al*, 2011; Grupp *et al*, 2013). Treatment was associated with remission of CLL and acute toxicities included macrophage activation syndromes and ‘cytokine storms’ as discussed below. All three CLL patients developed B cell aplasia, an expected consequence of anti-CD19 therapy. The investigators calculated effector:target cell ratios in these patients and estimated that *in vivo* T cell expansion greater than 1000-fold contributed to antileukaemic effects through serial killing effects. One of the two paediatric patients treated for ALL using autologous CAR19-modified T cells subsequently relapsed after 2 months with circulating CD34^+^ CD45^+dim^ CD19^−^ blasts, suggesting T cell-driven selective pressure allowing emergence of CD19^−^ populations. This type of tumour escape phenomenon highlights an important limitation of targeting a single antigen, but also provides a rationale for simultaneously targeting additional antigens, such as CD20 and CD22 in B cell malignancies. In relapsed myeloid malignancies CD30 and CD33 may provide suitable targets, but may also be problematic if there is associated depletion of myeloid progenitors and stem cell populations. Alternative tumour-associated antigens with wider applicability include Lewis Y (LeY), a difucosylated carbohydrate antigen, which has poorly defined functions, but is expressed on a wide range of malignancies, including certain forms of AML, but has only limited expression on normal tissue. Australian investigators coupled a CAR specific for LeY to cytoplasmic domains of CD28 and the CD3-ζ chain and have undertaken an initial safety study in patients with relapsed AML, where blasts were known to express LeY (Ritchie *et al*, 2013). Conditioning comprised fludarabine and four patients received up to 1·3 × 10^9^ T cells without significant toxicity. Cytogenetic remission was achieved in one patient and protracted remission in another. Interestingly, In^111^-labelled CAR T cells could be tracked *in vivo*, and were shown to traffic to the bone marrow using single photon emission computed tomography (SPECT) imaging. Experience form allogeneic transplantation suggests certain anatomical sites may provide sanctuary against immune-mediated anti-leukaemic effects and the effectiveness of cellular immunotherapy against extra-medullary tumours, including testicular relapse or central nervous system (CNS) disease, has yet to be elucidated. Nonetheless, EBV-specific T cells can track to extramedullary sites of EBV-lymphoproliferative disease (Heslop *et al*, 2010) and early trial reports suggest successful responses linked to the presence of CD19 CAR-modified T cells in cerebrospinal fluid in patients with CNS leukaemic relapse (Lee *et al*, 2013).

Whilst initial non-viral approaches based on plasmid transfer were inefficient, more recently trials have commenced at the MD Anderson Cancer Center in Texas using a Sleeping Beauty transposase system for first-in-man studies. Second generation CAR19 constructs, under the control of the human EF1α promoter and flanked by IR/DR transposition target sequences are being used in trials of autologous and alloegenic trials against B cell malignancies (Singh *et al*, 2014). The approach requires longer periods of *ex-vivo* culture compared to viral vector strategies, but is potentially more flexible for switching between different receptor and activation domain configurations.

## Conferring drug sensitivity and resistance

A number of trials have established the feasibility of using T cells engineered to be sensitive to particular drugs by inclusion of a ‘suicide gene’ mainly using the viral HSVTK gene linked to selection genes for antibiotic (e.g., Neomycin resistance) or magnetic bead selection (e.g., truncated forms of the low affinity nerve growth factor receptor) (Bonini *et al*, 1997; Tiberghien *et al*, 2001; Burt *et al*, 2003; Ciceri *et al*, 2007, 2009). Such cells have been administered following mismatched or halpoidentical transplantation and trials originally initiated two decades ago in Milan have reported improved immune reconstitution and control of GVHD by using ganciclovir in a number of subjects (Bonini *et al*, 1997; Tiberghien *et al*, 2001; Burt *et al*, 2003; Ciceri *et al*, 2007, 2009). To facilitate enrichment of suicide gene engineered cells, HSVTK has also been fused to a splice variant of human CD34, which enables rapid CliniMacs-based selection of transduced cells (Zhan *et al*, 2013). Although HSVTK encodes a virally-derived protein and immune responses against engineered cells have been detected, longer-term persistence has also been reported. An alternative inducible switch, based on a fusion of human caspase-9 and a variant human FK-binding protein has also been tested in pilot studies (Di Stasi *et al*, 2011). Here, suicide gene expression was linked to truncated CD19 for cell enrichment. Administration of a synthetic dimerizing drug activated caspase-mediated apoptosis of transduced cells. Similarly, expression of CD20, which can be targeted by rituximab, offers another potential avenue for targeted T cell elimination and is undergoing preclinical evaluation. Whilst these approaches have been developed primarily to allow infusion of polyclonal T cells populations whilst protecting against the risk of GVHD in allo-SCT, it is possible that they will also be included in vectors expressing novel antigen-specific receptors to safeguard against adverse effects in the autologous setting.

There have also been attempts to render T cells resistant to immunosuppressive agents, and such cells could have interesting niche applications. For example, adoptive transfer of EBV-specific T cells is an effective treatment for EBV-related lymphoproliferative disease but post-transplant immunosuppression following solid organ grafting can impair effective cellular responses. Calcineurin mutants have been designed to confer resistance to one or both of the commonly used immunosuppressants, tacrolimus and ciclosporin (Brewin *et al*, 2009; Ricciardelli *et al*, 2013) and proposals for pilot studies are being developed. Other approaches include rendering cells insensitive to steroids through nuclease-mediated disruption of the glucocorticoid receptor in T cells, and resistance against mycophenol phenolate through expression of mutant inosine monophosphate dehydrogenase (Yam *et al*, 2006; Sangiolo *et al*, 2007).

## Host preparation and managing adverse effects

Engraftment, persistence and efficacy of engineered T cells is enhanced by the use of preparative conditioning of patients. Agents, such as cyclophosphamide, fludarabine, pentastatin and low dose irradiation, or serotherapy are all capable of mediating lymphodepletion without myeloablation. Each approach has specific related toxicities, but all increase the risk of viral reactivation and other infectious complications. Nonetheless, in the conditioned lymphopenic host, T cells undergo homeostatic expansion and are less prone to the immunosuppressive effects mediated by host regulatory T cells. Furthermore, elimination of host lymphocytes reduces the risk of host-mediated immune responses against the engineered cells that could lead to rejection, especially if encoded receptors or other transgenes include non-human or novel protein sequences. There is currently little trial data comparing the intensity of conditioning required for effective responses, and larger studies with optimal vector-receptor configurations are required to determine the most suitable preparative regimens.

Post-infusion toxicities and side effects can be considered as immediate, infusion-related events and longer term expected or unexpected consequences. Early cytokine release syndrome (CRS) in CAR therapies has been related to multiple factors including γ-interferon (IFNγ, TNFα, IL1, IL2 and IL6, and may contribute to anti-leukaemic effects, but consequential inflammation and macrophage activation syndrome (MAS) can mimic sepsis with hypotension, fever rigours and vascular leak syndromes (Brentjens *et al*, 2013). Management has included supportive care and the use of methylprednisolone as well as a variety of monoclonal antibody therapies, including etanercept targeting TNFα and tocilizumab targeting the IL6 receptor. Differentiation from tumour lysis syndrome or bacterial sepsis may be complex, necessitating multiple therapeutic approaches. Davila *et al* (2014) recently proposed diagnostic criteria defining CRS based on the presence of fever, hypotension, hypoxia or the development of neurological complications in association with a 75-fold rise in serum levels of two relevant cytokines. It is plausible that the risk of precipitating CRS may be mitigated by reducing T cell doses, or by administration of multiple, smaller dose aliquots, and by dose escalation strategies. As more data accumulates it may also be possible to identify cytokine gene polymorphisms that predispose to exaggerated inflammatory responses. Finally, a longer-term and expected consequence of using CAR therapy targeting B cell antigens has been B cell aplasia and the need for replacement immunoglobulin therapy, although this is relatively straightforward to manage.

## Emerging tools for T cell engineering

**Fig 2:**
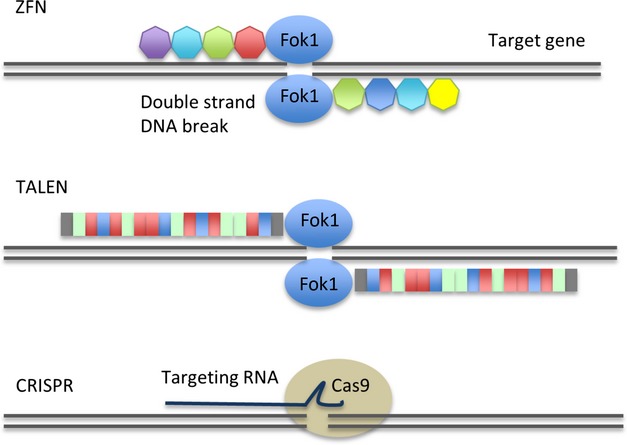
Molecular tools for gene editing are evolving and can mediate highly specific cleavage of double stranded DNA at an efficiency that is sufficient to be therapeutically useful. Zinc finger nucleases (ZFNs) are already being evaluated for disruption of the Human immunodeficiency virus co-receptor CCR5 in T cells. In common with alternative reagents based on transcription activator-like effector nucleases (TALENs), scission relies on dimerization of Fok1 endonucleases at defined genomic target sites. Recently, RNA-based DNA targeting reagents called clustered regularly interspaced short palindromic repeats (CRISPRs) have become available, and here, DNA cleavage is mediated by the enzyme Cas9. These reagents open up the possibility of designer T cells with multiple genetic modifications in combination with introduced receptors and safety/selection genes.

Emerging strategies are being investigated for gene-editing rather than conventional gene-addition approaches. Molecular reagents capable of mediating highly specific DNA cleavage using targeted nucleases are providing a variety of new possibilities. Examples include zinc finger nucleases (ZFNs) (Urnov *et al*, 2005; Lombardo *et al*, 2007), transcription activator-like effector nucleases (TALENs)(Miller *et al*, 2011) and clustered regularly interspaces short palindromic repeats (CRISPR) reagents (Jinek *et al*, 2012; Cong *et al*, 2013; Mali *et al*, 2013) and all are under investigation for a broad spectrum of gene therapy applications. Each have specialized DNA binding elements linked to nuclease enzymes capable of DNA scission (Fig2). One early example of clinical application is the recent use of ZFNs targeting the *CCR5* gene locus in trials aiming to disrupt expression of the HIV co-receptor in T cells from subjects with the infection (Tebas *et al*, 2014). Similarly, ZFNs and TALENs have been used to disrupt expression of endogenous T cell receptors in pre-clinical studies demonstrating the feasibility of generating T cells devoid of alloreactive TCRs by targeting the constant region of the TCR α and/or β chain (Provasi *et al*, 2012). Introduction of antigen-specific receptors into such cells could yield populations of ‘universal T cells’ that are suitable for infusion (following conditioning) into multiple HLA disparate recipients without the risk of GVHD (Torikai *et al*, 2012). Similar reagents are being developed to render T cells ‘invisible’ to a commonly used conditioning antibody, alemtuzemab. By disrupting CD52 expression, T cells rendered insensitive to depletion by alemtuzemab and could be infused immediately following serotherapy-conditioned transplant procedures, allowing exploitation of anti-viral or anti-leukaemia effects. Similarly, disruption of disparate HLA molecules on donor T cells may provide mitigation against host-mediated rejection (Torikai *et al*, 2013). For these applications, transient expression of DNA modifying nuclease reagents in T cells is desirable (rather than integrated, permanent gene insertion) and thus electroporation of mRNA and may be sufficient for therapeutic effect. The availability of next generation high throughput sequencing will facilitate screening for off-target genomic effects including undesirable chromosomal effects resulting in translocations, rearrangements or deletions, which necessitate careful characterization and monitoring.

## Delivering T cells beyond trials

Harvest, isolation, activation, transduction, expansion, selection and cryopreservation of patient-derived T cells to produce bespoke medicinal therapies requires notable infrastructure and expertise. Currently only a small number of centres worldwide can provide such therapies, and generally only as part of a structured clinical trial. Notable bottlenecks include limited vector production capacity for γRV or LV vector stocks and difficulties in scaling production for larger trials. Differences between European and other regulatory jurisdictions provides challenges for the implementation of multicentre clinical trials and ultimately the development of licensed medicinal products, although there is increasing dialogue aimed at addressing these issues. Wider deployment will also require a notable investment in addressing the logistics of harvesting, engineering and returning T cells to patients. There are, of course, established procedures and pathways for the harvest and infusion of bone marrow and peripheral blood stem cell products and networked transplant centres, which are well placed to deliver T cell therapies if manufactured, cryopreserved and shipped from a central processing hub. In recent months there has been considerable interest and investment from pharmaceutical companies aiming to establish strategic partnerships with leading research centres. Novartis, Celgene, Juno Therapuetcis, BlueBird Bio and others are all aiming to commercialize CAR therapies, and Kite Pharma and Adaptimmune are amongst companies pursuing αβTCR-based therapies (Humphries, 2013). There could be rapid dissemination of CAR19-based therapies if the logistical issues are addressed, and a broadening of early phase trials assessing a variety of other target antigens in both haematological and other malignancies should follow. The implications for health care purchasers and providers have yet to unfold, but pricing is likely to attract a premium if single episode cell therapies can offer the possibility of durable benefit or cure.

## Summary

Experience from studies using engineered T cells, including cells expressing chimaeric antigen-specific receptors, are providing proof of concept data for an emerging area of cellular therapy. Related vector platforms are being used to confer drug sensitivity and resistance, and emerging technologies are promising more sophisticated designer gene engineered T cells. Larger multi-centre trials will needed to determine which particular therapies offer the most effective interventions, and this in turn will require and number of technical and logistical hurdles to be addressed. Nonetheless, large investments are being proffered to accelerate clinical application, and prospects for T cell therapies look interesting.
